# Increased HIV Subtype Diversity Reflecting Demographic Changes in the HIV Epidemic in New South Wales, Australia

**DOI:** 10.3390/v12121402

**Published:** 2020-12-06

**Authors:** Francesca Di Giallonardo, Angie N. Pinto, Phillip Keen, Ansari Shaik, Alex Carrera, Hanan Salem, Christine Selvey, Steven J. Nigro, Neil Fraser, Karen Price, Joanne Holden, Frederick J. Lee, Dominic E. Dwyer, Benjamin R. Bavinton, Andrew E. Grulich, Anthony D. Kelleher, on behalf of the NSW HIV Prevention Partnership Project

**Affiliations:** 1 The Kirby Institute, The University of New South Wales, Sydney 2052, Australia; apinto@kirby.unsw.edu.au (A.N.P.); pkeen@kirby.unsw.edu.au (P.K.); ashaik@kirby.unsw.edu.au (A.S.); bbavinton@kirby.unsw.edu.au (B.R.B.); agrulich@kirby.unsw.edu.au (A.E.G.); akelleher@kirby.unsw.edu.au (A.D.K.); 2 Royal Prince Alfred Hospital, Sydney 2050, Australia; 3 HIV Reference Laboratory, Sydney 2010, Australia; alex.carrera@svha.org.au; 4 New South Wales Health Pathology-RPA, Royal Prince Alfred Hospital, Camperdown 2050, Australia; hanan.salem@health.nsw.gov.au (H.S.); frederick.lee@health.nsw.gov.au (F.J.L.); 5 Health Protection NSW, Sydney 2059, Australia; christine.selvey@health.nsw.gov.au (C.S.); steven.nigro@health.nsw.gov.au (S.J.N.); 6 Positive Life New South Wales, Sydney 2010, Australia; neilf@positivelife.org.au; 7 AIDS Council of NSW (ACON), Sydney 2010, Australia; kprice@acon.org.au; 8 NSW Ministry of Health, Sydney 2059, Australia; jo.holden@health.nsw.gov.au; 9 Sydney Medical School, University of Sydney, Sydney 2050, Australia; 10 New South Wales Health Pathology-ICPMR, Westmead Hospital, Westmead 2145, Australia; dominic.dwyer@sydney.edu.au

**Keywords:** HIV, non-B subtypes, transmission clusters, stage of infection, heterosexual transmission

## Abstract

Changes over time in HIV-1 subtype diversity within a population reflect changes in factors influencing the development of local epidemics. Here we report on the genetic diversity of 2364 reverse transcriptase sequences from people living with HIV-1 in New South Wales (NSW) notified between 2004 and 2018. These data represent >70% of all new HIV-1 notifications in the state over this period. Phylogenetic analysis was performed to identify subtype-specific transmission clusters. Subtype B and non-B infections differed across all demographics analysed (*p* < 0.001). We found a strong positive association for infections among females, individuals not born in Australia or reporting heterosexual transmission being of non-B origin. Further, we found an overall increase in non-B infections among men who have sex with men from 50 to 79% in the last 10 years. However, we also found differences between non-B subtypes; heterosexual transmission was positively associated with subtype C only. In addition, the majority of subtype B infections were associated with clusters, while the majority of non-B infections were singletons. However, we found seven non-B clusters (≥5 sequences) indicative of local ongoing transmission. In conclusion, we present how the HIV-1 epidemic has changed over time in NSW, becoming more heterogeneous with distinct subtype-specific demographic associations.

## 1. Introduction

The human immunodeficiency virus 1 (HIV-1) circulates globally as different genetically defined subtypes, with prevalence varying between different countries. Subtype C is most prevalent in Africa and subtype B is most prevalent in America, most of Europe, and Australia. In addition, over 98 circulating recombinant forms (CRFs) have been identified (Los Alamos National Lab (LANL) database (http://www.hiv.lanl.gov/). One of these, the circulating recombinant form 01_AE (CRF01_AE), is the most prevalent subtype in Southeast Asia [1]. A study by Angelis et al. showed that Thailand is the origin of the global CRF01_AE distribution, and this is consistent with Thailand’s popularity as a travel destination [2]. An increase in the proportion of CRF01_AE infections has recently been reported in the Philippines [3] and in Australia [4]. Furthermore, changes in subtype diversity are often related to migration patterns [5]. For example, a study from the USA reported an increase in non-B subtypes for numerous states that was associated with infections among foreign-born individuals [6]. Studies from Germany and Italy found that an increase in non-B subtypes was correlated to an increase in infections reported among individuals born in countries where the corresponding subtypes had a high prevalence [7,8]. Similarly, other studies found changes in subtype prevalence among individuals reporting differing transmission risk factors. For example, a study from China reported a decline in subtype B but an increase in CRF01_AE infections among individuals who reported heterosexual transmission [9]. Another study from Spain found an increase in non-B infections among men who have sex with men (MSM) [10]. These changes show that the HIV-1 epidemic is experiencing increased subtype diversity, which causes even further challenges for vaccine designs, but also HIV testing [11].

The prevalence of HIV-1 in Australia is low at 0.14%. The epidemic is characterised by a majority of infections occurring in MSM (63% of all HIV-1 notifications in 2017) and being of subtype B origin (62% in 2016) [4]. In addition, the epidemic is becoming more diverse. Between 2013 and 2017, a 11% decline in new infections associated with MSM transmission was reported, while during the same period a 10% increase in infections associated with heterosexual transmission was seen [4]. Further, differences in subtype prevalence are present between different Australian states; the subtype B prevalence varies between 59% in Western Australia and 82% in Queensland [12]. Most recently, South Australia reported a subtype B prevalence of only 36% for 2018 [13]. New South Wales (NSW) is the most populous state in Australia and accounts for approximately 30% of all HIV-1 infections annually [4]. We have reported previously an increase in CRF01_AE subtype prevalence from an average of 16% in the period 2004–2008 to 42% between 2014 and 2017 [14]. We have also shown the presence of local ongoing transmission for both subtype B and CRF01_AE ([15], accepted for publication [15]). However, it is unclear to what extent local ongoing transmission of other non-B subtype is present in NSW and to what extent demographic factors have changed in recent years for non-B infections. Thus, we report here: (i) the extent to which various demographic factors correlate with transmission patterns within different subtypes, (ii) the factors that are most or least similar between different subtypes, and (iii) how demographic associations have changed over time for different subtypes.

## 2. Materials and Methods

### 2.1. Ethics

Routinely collected sequence and demographic data on all newly notified HIV-1 infections are linked and irreversibly de-identified to enable public health research in NSW as previously described [14]. Ethics Committee approval was obtained through the NSW Population and Health Services Research Ethics Committee and the ACON Research Ethics Review Committee (RERC) [AU RED Reference: HREC/15/CIHS/38, Cancer Institute NSW reference number: 2015/08/605]. The Ethics Committee granted a waiver for consent of the individual to use their health information.

### 2.2. Sequence Phylogeny and Identification of NSW-Specific Clusters

A total of 2364 sequences of the reverse transcriptase (RT) were retrieved from the local database that includes all HIV-1 sequences in NSW: these represent sequences from new HIV notifications reported between 2004 and 2018. HIV-1 subtype was determined using the Stanford HIV subtyping tool and confirmed via a phylogenetic tree estimated using RAxML v8.2.12 [16,17]. The sequences fell into subtypes B, CRF01_AE, C, A, and multiple individual other subtypes and CRFs. The same NSW sequences were then combined with global sequence data retrieved from the HIV LANL database as described elsewhere [18]. In short, NSW HIV-1 RT nucleotide sequences were compared against global HIV-1 sequences via BLASTN to retrieve most similar sequence data to be used as background. Sequences were aligned using MAFFT L-INS-I [19] and the alignment was inspected in Geneious 11.1.3 (https://www.geneious.com) for accuracy. The final alignment consisted of 6300 sequences and 628 nt in length. A phylogenetic tree was estimated using FastTreeMP v2.1.10 [20]. NSW-specific clusters were defined as sequence nodes containing 100% of NSW sequences (monophyletic). Although, it is common to use partial pol including protease for HIV-1 sequence we decided to use the RT only as one of the laboratories performing standard diagnostic testing does not process the protease any longer. Thus, we could assure complete coverage for all sequences analysed. RT alignment length has been used for phylogenetic analysis and has sufficient signal for an accurate phylogenetic analysis [21].

### 2.3. Demographic Factors

Demographic data included (i) sex: male, female; (ii) region of birth: Australia, not Australia; (iii) transmission risk factor: MSM (men who have sex with men), heterosexual, people who inject drugs (PWID), other; (iv) age groups: 0–19 years, 20–29 years, 30–39 years, 40–49 years, 50+ years; and (v) residential area based on categories of postcodes according to the proportion of adult gay men: <5%, 5–19.9%, and ≥20% [22]. Disease stage data included stage of infection at diagnosis. Infection stage categories were adapted from the NSW Ministry of Health definitions [23]: early = evidence of an HIV-1 infection acquired within 12 months of diagnosis or CD4+ T-cell count > 500 cells/mm^3^, CD4+ T-cell count 350–499 cells/mm^3^, CD4+ T-cell count 200–349 cells/mm^3^, and advanced = CD4+ T-cell count < 200 cells/mm^3^ or AIDS defining illness in absence of early diagnosis. Chi-square tests of independence for the association of demographic data with HIV subtype were performed in *R* v3.6.2 [24] using the *gplots* and *corrplots* packages [25,26] and linear regression and correlation coefficient were calculated in MATLAB^®^ v2020a and regarded a significant with *p* values < 0.05 [27]. To ensure the individuals’ data privacy, demographic data containing <5 sequences per year analysed was will not be shown. Instead, proportion of sequence data rather than total number is shown for demographic comparisons, though, these are not independent of each other. In addition, demographic changes over time were analysed for the time period 2012–2018 due to the limited sequence data available in previous years (<5 sequences for non-B subtypes per year analysed).

### 2.4. Data Availability

A random subset of 10% of the NSW sequences analysed here is available via NCBI under the GenBank accession numbers MW246250-MW246484. This will prevent the potential identification of sequence networks and ensure the individuals’ data privacy.

## 3. Results

### 3.1. Subtype B and CRF01_AE Dominate the HIV-1 Epidemic in New South Wales

**Figure 1:**
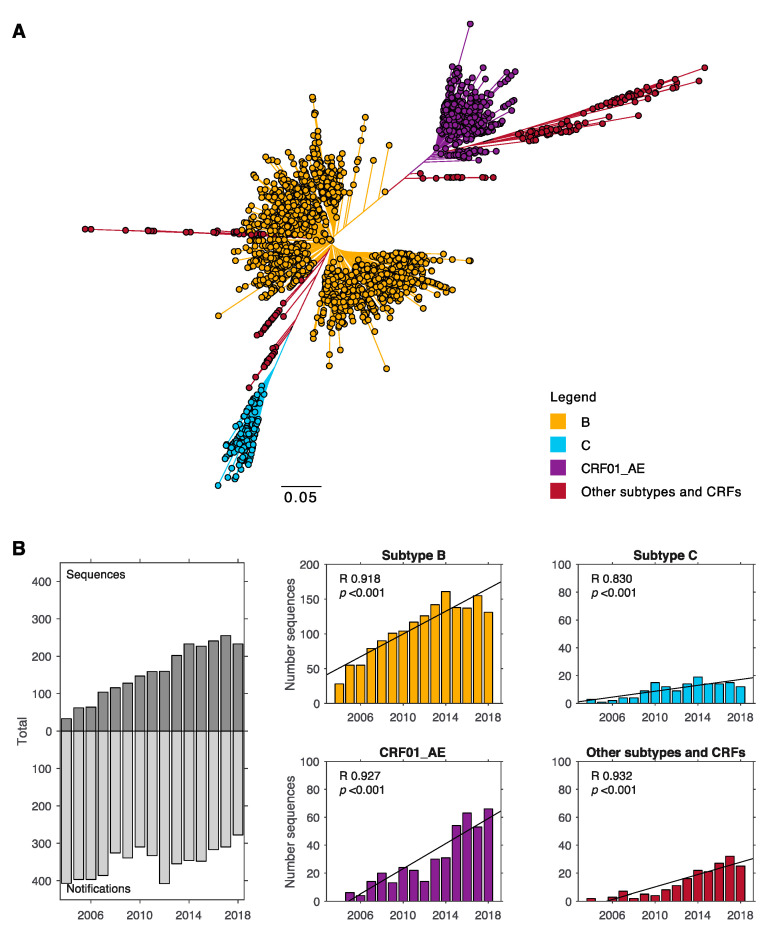
HIV-1 subtype diversity in New South Wales (NSW) for the past 14 years. (**A**) Maximum likelihood tree for HIV-1 sequences in NSW. Reverse transcriptase sequences sampled between 2004 and 2018 were used to estimate a tree phylogeny. Branch length indicates nucleotide substitutions per site. (**B**) Left panel: Number of sequences used in this analysis compared to the number of new HIV-1 infections notified in NSW. Other panels: number of sequences for different subtypes across time and linear regression with correlation coefficient (R) and *p* values indicated. Yellow = subtype B, cyan = subtype C, purple = CRF01_AE, and red = other subtypes and CRFs (including CRF02_AG, CRF07_BC, CRF60_BC, CRF44_BF, CRF06_cps, CRF12_BF, CRF33_01B, CRF35_AD, CRF77_cpx, and CRF55_01B, A, F, D, G).

In NSW, the majority of HIV-1 infections are of subtype B origin. Between 2004 and 2018, 70% of sequences were of subtype B, 18% of CRF01_AE, and 6% of subtype C origin (Figure 1A). The remaining sequences included subtype A (2%), and subtypes F, G, and D (1%), and other CRFs (2%). Data used in our analysis included only sequences that were linked to a newly notified case of HIV, and thus had demographic data available. The proportion of sequences that were able to be linked to a notified case increased over time from 34% in 2004 to 51% in 2011 and 83% in 2018. Thus, the number of sequences included in the analysis here was higher in 2018 (*n* = 233) compared to 2006 (*n* = 64) even though the annual number of new HIV-1 notifications in NSW has been declining (*n* = 397 in 2006, *n* = 278 in 2018) (Figure 1B) [23]. The proportion of sequences that could be linked to meta data did not differ across subtypes B, C, and other subtypes and CRFs (*p* = 0.57, linked proportions of between 57 and 60%). However, a slightly higher proportion of sequences could be linked for CRF01_AE (63%, *p* < 0.01). Due to this improved data collection, the number of sequences increased significantly over time for all subtypes (Figure 1B). Most notably, for CRF01_AE from 0 to 66 sequences between 2004 and 2018.

### 3.2. Subtype C is Strongly Associated with Hetereosexual Transmission

The subtypes differed significantly in their associated demographics (Table 1, Figure S1A). First, subtype B dominated the data set with 68% of sequences deriving from infections of this subtype, and for almost all demographics the majority of infections were subtype B. Two exceptions were infections among females and individuals reporting heterosexual transmission, for which only 27% and 35% where of subtype B origin, respectively. Overall, subtype B infections were positively associated with individuals that were male, born in Australia, 40–49 years old, living in postcodes with a gay male population of ≥20%, reporting MSM transmission as their risk factor, or were diagnosed during the early stage of infection (*p* < 0.001). Non-B subtypes were positively associated with infections among individuals that were female, not born in Australia, <30 years of age, living in postcodes with a gay male population of <5%, reporting heterosexual transmission, or being diagnosed during the advanced stage of infection (*p* < 0.001).

Demographics also differed within non-B subtypes (Table 1, Figure S1A). For non-B infections, CRF01_AE was the dominant subtype, making up 56% of all non-B infections. Similar to subtype B, CRF01_AE accounted for the majority of non-B infections among individuals that were male, born in Australia, >20 years old, living in postcodes with a gay male population of >5%, or reporting MSM transmission (Table 1). Thirty-eight percent of infections among females were subtype C (*p* < 0.001, Figure S1A). In our study, we found that a large proportion of people with non-B subtypes reported heterosexual contact (53%, 29%, and 33% for subtype C, CRF01_AE, and other subtypes and CRFs, respectively). In fact, heterosexual transmission was positively associated with all non-B subtypes (*p* < 0.001). In addition, significant differences were also found for age (*p* < 0.05), postcode according to gay male population (*p* < 0.001), transmission risk factor (*p* < 0.001), and stage of infection (*p* < 0.01) (Figure S1). Subtype C infections were also more likely to be among individuals that were <20 years of age and 40–49 years old, living in postcodes with a gay male population of <5%, or reported heterosexual transmission. CRF01_AE infections had a positive association with individuals that were male, >50 years old, living in a postcode with a gay male population of 5–19%, reported MSM transmission, or were diagnosed during the advanced stage of infection. All other subtypes and CRFs combined had a positive association with individuals that were 20–29 years old or were diagnosed during the early stage of infection.

### 3.3. Changes in Associations between Demographics and Subtype between 2009 and 2018

**Figure 2:**
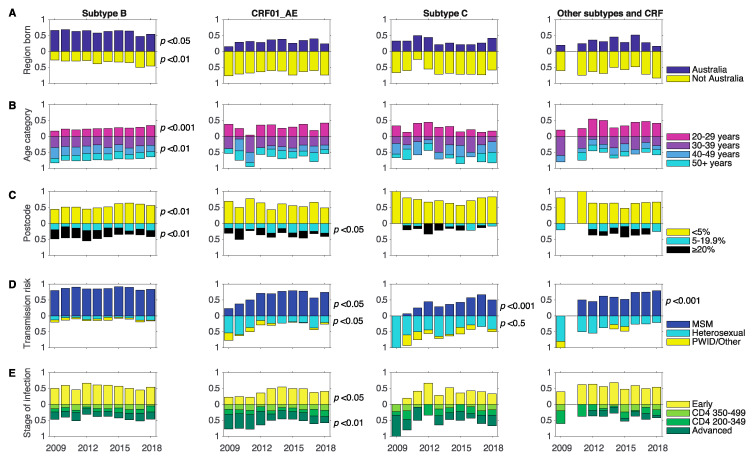
Time-specific changes in demographics. Proportion of sequences from infections among individuals with different demographics are shown for the time span of 2009–2018 for subtype B, C, CRF01_AE, and other subtype and CRFs. *p* values showing significant changes over time are shown next to the panel. (**A**) region born: Australia (blue), not-Australia (yellow); changes over time were significant for subtype B. (**B**) Age category: 20–29 years old (pink), 30–39 years old (purple), 40–49 years old (blue), 50+ years (cyan); changes were significant for subtype B 20–29 years old and 40–49 years old). (**C**) Postcode category according to proportion of gay male population: <5% (yellow), 5–19.9% (cyan), ≥20% (black); changes were significant for CRF01_AE 5–19.9%. (**D**) Transmission risk factor: MSM (blue), heterosexual (cyan), PWID/Other (yellow); changes were significant for subtype C MSM and heterosexual, CRF01_AE MSM, and other subtypes and CRFs MSM. (**E**) Stage of infection: early (green), CD4 350–499 (light green), CD4 200–349 (darker green), and advanced (dark green); changes were significant for CRF01_AE advanced. *p* values for linear regression are indicated for the following linear regression correlation coefficients: Subtype B region born Australia (R = −0.670, *p* < 0.05) and not Australia (R = 0.832, *p* < 0.01), age category 20–29 years old (R = 0.927, *p* < 0.001) and 40–49 years old (R = −0.854, *p* < 0.01), postcode < 5% (R = 0.781, *p* < 0.01) and postcode ≥20% (R = −0.816, *p* < 0.01); CRF01_AE postcode 5–19.9% (R = 0.703, *p* < 0.05), transmission risk factor MSM (R = 0.724, *p* < 0.05) and PWID/Other (R = −0.659, *p* < 0.05), stage of infection early (R = 0.649, *p* < 0.05) and advanced (R = −0.860, *p* < 0.01); subtype C transmission risk factor MSM (R = 0.896, *p* < 0.001) and heterosexual (R = −0.752, *p* < 0.05); and other subtypes and CRFs transmission risk factor MSM (R = 0.885, *p* < 0.001).

We investigated changes over time between subtypes and different demographics for the time period 2009–2018 as it contained sufficient data for all subtypes analysed. Between 2009 and 2018, the changes in subtype-specific demographics over time differed for subtype B and non-B infections. This was most notable for region of birth and transmission risk factors (Figure 2). For subtype B, we found significant changes in three of the five demographics analysed. We observed a decrease in the proportion of B infections from 66% to 54% among those born in Australia (*p* < 0.05), but an increase from 27% to 46% among those not born in Australia (*p* < 0.01). We also observed a decrease in infections among individuals aged 20–29 years (*p* < 0.001) and in parallel a decrease among those aged 40–49 years (*p* < 0.01). No changes were found for transmission risk factor and stage of infection. For CRF01_AE we found significant changes over time for transmission risk, and stage of infection. We found an increase in infections among individuals reporting MSM transmission combined with a decrease in infections among individuals reporting PWID or other as a transmission risk factor (*p* < 0.05). Further, we observed an increase in infections among individuals diagnosed during the early stage (*p* < 0.05) combined with a decrease in infections among individuals diagnosed during the advanced stage (*p* < 0.01). We also found an increase in infections among MSM for subtype C and other subtypes and CRFs (*p* < 0.001) and this was combined with a decrease in infections among heterosexuals for subtype C (*p* < 0.05). No other demographics showed significant changes over time for subtype C and other subtypes and CRFs.

A study by Callander et al. showed the vast discrepancy in the proportion of gay men and lesbian women living in different residential areas in NSW [22]. They showed that 2.7% of postcodes were highly populated by gay men and lesbian women, i.e., ‘gayborhoods’. They also identified postcodes with an estimated gay male population of ≥20%. Here, we used this differentiation to identify changes in HIV-1 diversity within and outside these postcodes. Interestingly, for subtype B, we found a decrease from 29% to 19% in the proportion of infections among individuals living in postcodes with a gay male population of ≥20% (*p* < 0.01). However, we found an increase from 45% to 57% in the proportion of infections individuals living in postcodes with a gay male population of <5% (*p* < 0.01). We also found an increase from 15% to 30% for CRF01_AE infections among individuals living in postcodes with a gay male population of 5–19.9% (*p* < 0.05).

### 3.4. Demographic Differences in Clusters and Singleton Sequences

**Figure 3:**
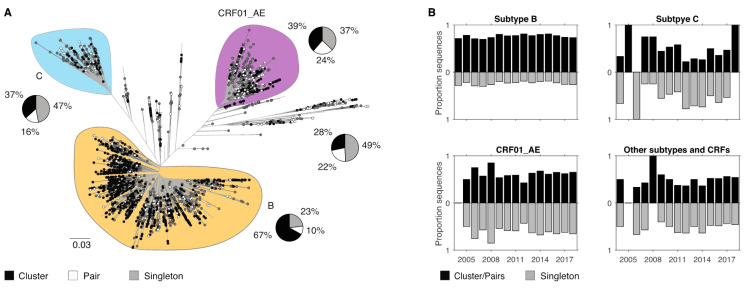
HIV-1 clusters in NSW for different subtypes. (**A**) Unrooted maximum likelihood tree from NSW and global reverse transcriptase sequences. Branch length indicates nucleotide substitutions per site. Circle at tips indicate sequences from NSW, while sequences without circles are global sequences. Clades representing subtype B, C, and CRF01_AE are colored. Tip circles are colored according to being part of a cluster (black), sequence pair (white), or a singleton (gray). The proportion of sequences associated with each cluster type or singleton is shown as a pie chart for the four subtype groups analysed. (**B**) Proportion of sequences from infections associated with cluster and pairs or being singletons are shown for the four subtype categories across time.

Patterns of HIV-1 transmission were assessed by investigating sequence clusters for all subtypes. Overall, 232 sequence clusters and 165 sequence pairs were identified. For subtype B, 67% of all infections were associated with a cluster (>3 sequences) and 10% were associated with a sequence pair (Figure 3A). In contrast, for people with subtype C, CRF01_AE, and other subtypes and CRFs a much lower proportion of infections were associated with a cluster (37%, 39% and 28%, respectively). These latter three subtype categories were characterised by a high frequency of singleton sequences (47%, 37%, 49% for subtype C, CRF01_AE, and other subtypes and CRFs, respectively). The proportion of infections that were associated with a cluster or not was also compared over time, but no changes were found for either subtype or CRF (Figure 3B).

Finally, we compared demographics for infections in clusters and pairs and those that were singletons Table S1). Interestingly, no differences were found for any of the non-B subtypes with similar proportion of infections being either a cluster/pair or singletons for each of the demographics analysed. However, we found significant differences for subtype B (Figure S1B). In concordance with our previous study, we found a strong positive correlation for infections among individuals not born in Australia (*p* < 0.001), as well as among heterosexuals, for being singletons (*p* < 0.01). Further, infections from notifications during the advanced stage were more likely to be singletons (*p* < 0.05). In addition, we found here that subtype B infections among individuals 50 years and older were also more likely to be singletons (*p* < 0.05).

## 4. Discussion

We report here on the growing diversity of HIV-1 in NSW. We show how non-B subtypes have increased over time, particularly among individuals reporting MSM as a transmission risk factor. We also show how subtype-specific demographics differ in general between subtype B and non-B subtypes, which also changed over time. In addition, we showed the importance of sequence data for epidemiological analysis as notification data alone do not represent the changes in genetic variability within an epidemic.

Overall, we found significant differences between subtype B and non-B subtypes for all demographics analysed. Subtype B was strongly associated with male individuals and those born in Australia, while non-B subtypes were associated with female individuals and those not born in Australia. The strongest positive association was found for subtype C being among female individuals (*p <* 0.001). A large proportion of non-B subtypes infections among females has also been reported in the adjoining state, Victoria. In their study, Chibo et al. identified 85% of infections among female individuals reported in 2010 being of non-B origin [28]. In addition, 62% of infections of other subtypes and CRFs were among individuals 20–29 years old and not born in Australia. This positive association between young individuals and non-B HIV infection could in part be explained by the relatively high number of international students completing higher education in Australia (~30% of all higher education students were international in 2018) [29]. Further, NSW Health reported a 33% increase in late diagnosis, i.e., diagnosis made >12 months after transmission, among MSM not born in Australia and concludes that these infections are likely to be acquired outside Australia [23]. However, in our study we found a decrease in the proportion of infections notified during the advanced stage only for CRF01_AE from 46% to 20% between 2009 and 2018 (*p* < 0.01) but found no changes over time for any other subtype. The decline in advanced diagnosed for CRF01_AE infections was combined with an increase in diagnoses during the early stage of infection, which indicates an increase in early testing for individuals harboring CRF01_AE.

We observed an overall increase in infections among MSM for all non-B subtypes over time; for subtype C (0% to 50%), for CRF01_AE (23% to 74%), and for other subtypes and CRFs (0% to 79%). This means that the observed decline in HIV-1 infections in NSW [23] is driven by a decrease in subtype B infections among MSM born in Australia only, while the number of non-B infections has not changed. However, at this point it is unclear what has caused the dichotomy in number of infections between subtype B and non-B among individuals born in Australia and those not born in Australia, as well as among individuals reporting MSM and heterosexual transmission risk. In Australia, infections with non-B subtypes are often associated with migration or travel and are thus more likely to have been acquired elsewhere. Thus, individuals acquiring these infections might not have been exposed to local prevention strategies, subsequently their numbers would not have changed over time. However, HIV-1 acquisition outside of Australia does not explain the observed increase in subtype B infections among individuals not born in Australia, which could indeed represent local transmission.

Notably, we found changes for infections in individuals living in postcodes with a high or low proportion of gay male population. For subtype B, we found an increase in infections among individuals living in postcodes with a gay male population of <5% and a parallel decrease for infections among individuals living in postcodes with a gay male population ≥20%, indicating a shift from ‘gayborhoods’ to suburbs with low proportion of gay men. Our findings are consistent with the study by Grulich et al. that reported a 52% decline in HIV-1 notifications within postcodes in Sydney, NSW, where >10% of resident men identified as gay compared to 7% in other residential areas [30]. Their study investigated the impact of pre-exposure prophylaxis (PrEP) rollout in NSW on the number of new HIV-1 infections in the state and reported an overall decline of 25% among MSM. However, the study also showed that this decline was lower in postcodes with a gay male population of <10%. Another study found a lower PrEP uptake in gay and bi-sexual men aged 25 years and younger in NSW [31]. Taken together, these variable trends across different subtypes and demographics indicate potential variable penetration of public health initiatives, such as those to increase testing rates or to increase access to PreP in at risk populations.

Finally, we quantified transmission clusters and found some clusters containing <5 sequences which are indicative of ongoing local transmission. Such subtype B and CRF01_AE clusters have been extensively described elsewhere ([15], accepted for publication) and will not be discussed here. In the current study, we found three subtype C clusters (*n* = 7 infections, *n* = 5, *n* = 5), one CRF02_AG cluster (*n* = 7), one subtype A cluster (*n* = 6), and one CRF60_BC cluster (*n* = 5). However, only two of these clusters contained infections sampled in 2017 or 2018 that are indicative of recent local transmission. In addition, we found that while subtype B contained overall a majority of infections associated with clusters (67%), non-B subtypes contained a majority of infections that were either singletons or pairs. This means that non-B subtypes rarely lead to ongoing transmission in NSW.

This study had a number of limitations. Most notably, molecular data only include sequences from NSW, thus, we lack data from other states in Australia and might miss inter-state transmission. Further, we were only able to use the *reverse transcriptase* gene for phylogenetic analysis which might limit the depth of genetic clustering found here. However, we are using global data as background and thus clusters are defined by being more closely related to each other than to sequence data from other regions [32]. We also use a very conservative threshold of 100% NSW-specific sequences being part of a cluster and believe that both these parameters increase the sensitivity of cluster analysis. We also lack some demographics, e.g., time since arrival in Australia that would improve associations of HIV-1 acquisition outside Australia and potential transmission in NSW.

## 5. Conclusions

In conclusion, we found significant differences in the demographic characteristics for newly diagnosed patients with different HIV subtypes. People with non-B subtypes were mostly foreign born, people with subtype C were mostly heterosexual, and people with other subtypes and CRFs were mostly 20–29 years old. Lastly, though limited, ongoing local transmission for non-B subtypes was observed in NSW. These data may assist with targeted prevention interventions and suggests that ongoing surveillance of HIV-1 molecular data is needed to continue to inform strategies aimed at virtual elimination of HIV in NSW.

## Figures and Tables

**Table 1:** Subtype-specific demographics. Proportion of infections associated with different demographics are shown for subtype B and all non-B subtypes, as well as individual non-B; subtype C, CRF01_AE and other subtypes and CRFs combined. Proportions are shown with the total number in brackets. Chi-square *p* values are shown in Figure S1A.

Demographic (*n*)	Subtype B	All non-B	CRF01_AE	Subtype C	Other Subtypes and CRFs
Sex					
Male (1825)	0.73 (1330)	0.27 (495)	0.60 (298)	0.16 (80)	0.24 (117)
Female (139)	0.27 (37)	0.73 (102)	0.36 (37)	0.38 (39)	0.25 (26)
No data (397)	0.63 (252)	0.37 (145)	0.52 (76)	0.19 (28)	0.28 (41)
Region Born					
Australia (1249)	0.81 (1007)	0.19 (242)	0.56 (135)	0.18 (44)	0.26 (63)
Not Australia (1012)	0.53 (533)	0.47 (479)	0.57 (271)	0.19 (93)	0.24 (115)
No data (103)	0.77 (79)	0.23 (24)	0.33 (8)	0.42 (10)	0.25 (6)
Age Category					
<20 years (37)	0.51 (19)	0.49 (18)	0.39 (7)	0.33 (6)	0.28 (5)
20–29 years (632)	0.63 (400)	0.37 (232)	0.53 (124)	0.16 (36)	0.31 (72)
30–39 years (742)	0.70 (518)	0.30 (224)	0.58 (129)	0.20 (44)	0.23 (51)
40–49 years (534)	0.74 (394)	0.26 (140)	0.50 (70)	0.27 (38)	0.23 (32)
50+ years (419)	0.69 (288)	0.31 (131)	0.64 (84)	0.18 (23)	0.18 (24)
Postcode Category according to the Proportion of Adult Gay Men					
<5% (1281)	0.65 (827)	0.35 (454)	0.50 (228)	0.24 (109)	0.26 (117)
5–19.9% (425)	0.67 (284)	0.33 (141)	0.72 (101)	0.08 (11)	0.21 (29)
>20% (538)	0.80 (430)	0.20 (108)	0.58 (63)	0.13 (14)	0.29 (31)
No data (120)	0.65 (78)	0.35 (42)	0.52 (22)	0.31 (13)	0.17 (7)
Transmission Risk Factor					
MSM (1815)	0.76 (1385)	0.24 (430)	0.62 (266)	0.12 (50)	0.27 (114)
Heterosexual (393)	0.35 (137)	0.65 (256)	0.46 (118)	0.30 (78)	0.23 (60)
PWID/Other (156)	0.62 (97)	0.38 (59)	0.51 (31)	0.32 (19)	0.17 (10)
Stage of Infection at Diagnosis					
Early (1224)	0.74 (903)	0.26 (321)	0.52 (168)	0.17 (53)	0.31 (100)
CD4 350 to 499 (278)	0.64 (179)	0.36 (99)	0.60 (59)	0.17 (17)	0.23 (23)
CD4 200 to 349 (278)	0.62 (172)	0.38 (106)	0.52 (55)	0.24 (25)	0.25 (26)
Advanced (495)	0.62 (309)	0.38 (186)	0.63 (118)	0.22 (41)	0.15 (27)
No data (89)	0.63 (56)	0.37 (33)	0.42 (14)	0.33 (11)	0.24 (8)

MSM: men who have sex with men; PWID: people who inject drugs.

## Supplementary Materials

The following are available online at https://www.mdpi.com/1999-4915/12/12/1402/s1, Figure S1: Correlation plot showing Chi-square statistics for demographic factors and different subtypes, Table S1: Subtype-specific demographics for sequences associated with clusters and singletons.
