# Supplementary material for: Increased HIV Subtype Diversity Reflecting Demographic Changes in the HIV Epidemic in New South Wales, Australia
Source: Viruses. 2020 Dec 6;12(12):1402. doi: 10.3390/v12121402 (PMC7762219; doi:10.3390/v12121402)
Supplement: Supplementary file 1 [file viruses-12-01402-s001.pdf]

viruses

MDPI

Table S1. Subtype-specific demographics for infections associated with clusters and singletons. Proportion of infections associated with different demographics are shown for individual subtypes and subtype groups analyzed. Chi-Square *p* values are indicated.

	**Subtype B (n=1619)**		**Non-B (n=745)**		**CRF01_AE (n=414)**		**Subtype C (n=147)**		**Other (n=184)**	
	**Cluster/Pairs**	**Singletons**	**Cluster/Pairs**	**Singletons**	**Cluster/Pairs**	**Singletons**	**Cluster/Pairs**	**Singletons**	**Cluster/Pairs**	**Singletons**
	**0.77 (1245)**	**0.23 (374)**	**0.58 (432)**	**0.42 (313)**	**0.63 (261)**	**0.37 (153)**	**0.53 (78)**	**0.47 (69)**	**0.51 (93)**	**0.49 (91)**
Sex										
Male	0.64 (1030)	0.19 (300)	0.39 (287)	0.28 (208)	0.46 (190)	0.26 (108)	0.27 (40)	0.27 (40)	0.31 (57)	0.33 (60)
Female	0.02 (29)	<0.01 (8)	0.08 (63)	0.05 (39)	0.06 (25)	0.03 (12)	0.15 (22)	0.12 (17)	0.09 (16)	0.05 (10)
Region born										
Australia	0.50 (805)	0.12 (202)	0.08 (158)	0.11 (84)	0.06 (94)	0.10 (41)	0.18 (26)	0.12 (18)	0.21 (38)	0.14 (25)
Not Australia	0.23 (379)	0.10 (154)	0.35 (262)	0.29 (217)	0.39 (162)	0.26 (109)	0.32 (47)	0.31 (46)	0.29 (53)	0.34 (62)
Age category										
20-29 years	0.19 (313)	0.05 (87)	0.17 (129)	0.14 (103)	0.17 (72)	0.13 (52)	0.14 (20)	0.11 (16)	0.20 (37)	0.19 (35)
30-39 years	0.26 (416)	0.06 (102)	0.17 (123)	0.14 (101)	0.18 (75)	0.13 (54)	0.18 (26)	0.12 (18)	0.12 (22)	0.16 (29)
40-49 years	0.19 (301)	0.06 (93)	0.12 (86)	0.07 (54)	0.12 (50)	0.05 (20)	0.14 (20)	0.12 (18)	0.09 (16)	0.09 (16)
50+ years	0.12 (202)	0.05 (86)	0.11 (83)	0.06 (48)	0.14 (60)	0.06 (24)	0.05 (7)	0.11 (16)	0.09 (16)	0.04 (8)
Postcode category according to the proportion of adult gay men										
<5%	0.39 (632)	0.12 (195)	0.36 (265)	0.25 (189)	0.35 (145)	0.20 (83)	0.37 (55)	0.37 (54)	0.35 (65)	0.28 (52)
5-19.9%	0.14 (227)	0.04 (57)	0.10 (78)	0.08 (63)	0.14 (59)	0.10 (42)	0.05 (8)	<0.02 (<5)	0.05 (11)	0.10 (18)
>20%	0.21 (333)	0.06 (97)	0.09 (67)	0.06 (41)	0.10 (42)	0.05 (21)	0.06 (9)	0.03 (5)	0.09 (16)	0.08 (15)
Transmission risk factor										
MSM	0.67 (1078)	0.19 (307)	0.32 (237)	0.26 (193)	0.38 (159)	0.26 (107)	0.18 (27)	0.16 (23)	0.28 (51)	0.34 (63)
Heterosexual	0.06 (91)	0.03 (46)	0.21 (160)	0.13 (96)	0.20 (81)	0.09 (37)	0.29 (43)	0.24 (35)	0.20 (36)	0.13 (24)
PWID/Other	0.05 (76)	0.01 (21)	0.05 (35)	0.03 (24)	0.05 (21)	0.02 (9)	0.05 (8)	0.07 (11)	0.03 (6)	<0.02 (<5)
Stage of infection at diagnosis										
Early	0.45 (734)	0.10 (169)	0.26 (192)	0.17 (129)	0.27 (112)	0.14 (56)	0.19 (28)	0.17 (25)	0.28 (52)	0.26 (48)
CD4 350 to 499	0.09 (138)	0.03 (41)	0.08 (59)	0.05 (40)	0.08 (35)	0.06 (24)	0.07 (11)	0.04 (6)	0.07 (13)	0.05 (10)
CD4 200 to 349	0.08 (129)	0.03 (43)	0.08 (60)	0.06 (46)	0.08 (35)	0.05 (20)	0.09 (13)	0.08 (12)	0.07 (12)	0.08 (14)

MSM men who have sex with men

PWID people who inject drugs

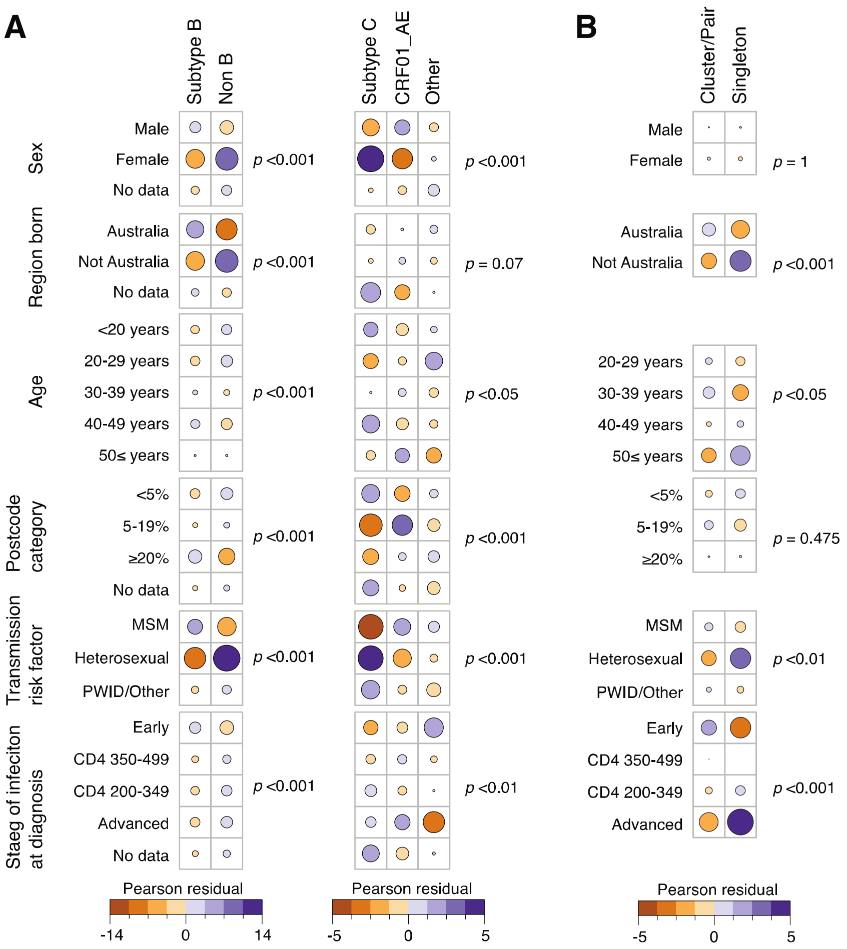

Figure S1. Correlation plot showing Chi-square statistics for demographic factors and different subtypes. The Pearson residual value is shown for each association (cell). purple = positive association, yellow = negative association. More intense colours and larger squares equal stronger contribution to the overall Chi-square score. **A** Demographics for B compared to non B infections (left panel) and between non-B infections (right panel). **B** Demographics for subtype B infections in clusters compared to singletons. MSM = men who have sex with men, PWID = person who inject drugs.
